# Optimizing Operating Parameters of Electric Ultra-Low Volume Sprayer with Slightly Acidic Electrolyzed Solution for Efficient Virucidal Activity on Environmental Surfaces

**DOI:** 10.3390/ijerph181910183

**Published:** 2021-09-28

**Authors:** Hae-Won Lee, So-Ra Yoon, Hyeyeon Song, Boyeon Park, Ji-Hyoung Ha

**Affiliations:** Hygienic Safety and Analysis Center, World Institute of Kimchi, Gwangju 61755, Korea; lhw0875@wikim.re.kr (H.-W.L.); sorayoon@wikim.re.kr (S.-R.Y.); danbihy@wikim.re.kr (H.S.); boyeonpark@wikim.re.kr (B.P.)

**Keywords:** human norovirus, optimization, response surface methodology, slightly acidic electrolyzed water, spray disinfection

## Abstract

Recently, and considering the COVID-19 pandemic, there has been a growing consensus that the disinfection of surfaces contaminated with pathogenic viral particles is essential. Chemical disinfectant sprays are effective at preventing the spread of infectious human noroviruses (Hu-NoVs) in healthcare and public areas. We assessed the virucidal activity of slightly acidic electrolyzed water (SAEW) spray on fomite surfaces. A multivariate statistical assessment that combined a response surface methodology (RSM) and a Box–Behnken design (BBD) was performed to define the optimal parameters of, and correlations among, experimental conditions. Spraying SAEW disinfectant (oxidation-reduction potential: 1123 mV, pH range: 5.12, available chlorine concentration: 33.22 ppm) resulted in the successful decontamination of Hu-NoV, with a 4-log reduction in viral particles on polyvinyl chloride, stainless steel, ceramic tile, and glass surfaces. Our experimental data revealed optimized treatment conditions for decontaminating Hu-NoV GI.6 and GII.4, using the numerical multiple optimized method (spraying rate: 218 mL/min, spraying time: 4.9 s, spraying distance: 0.9 m). These findings offer significant insights for designing optimal strategic control practices to prevent infectious disease, particularly Hu-NoV, transmission.

## 1. Introduction

Human noroviruses (Hu-NoVs), characterized by high infectivity among humans, short-term immunity, tenacious environmental persistence, and high viral load excretion, cause sporadic cases of nonbacterial gastroenteritis and represent the most significant etiologic agent of human epidemics, regardless of age or sex, and likely owing to their high-transmissibility across industrialized nations [1]. Moreover, the Centers for Disease Control and Prevention (CDC) reported that, annually, Hu-NoV infections cause an estimated 200,000 deaths, with more than 590,000,000 cases worldwide [2].

Historically, gastrointestinal viral pathogens have been shown to be readily transmitted via waterborne or environmental fomite routes, direct person-to-person transmission, or the fecal–oral route [3]. However, more recently, airborne viral pathogens, including those in aerosolized droplets (e.g., Hu-NoVs) have been proven to be particularly hazardous. In fact, previous studies have asserted that aerosolized Hu-NoV particles, produced by carrier patients, represent a significant source of transmission [4,5]. Bonifait et al. demonstrated that viral droplets can be aerosolized from infected gastrointestinal bodily fluids, including feces or vomit, which can become deposited in the upper respiratory tract during inhalation and subsequently swallowed; alternatively, these aerosolized particles can become attached to the surface of environmental fomites [6]. In the case of infectious Hu-NoVs, viral particles on environmental surfaces often serve as the epicenter of secondary cross-contamination. In fact, several studies have investigated the Hu-NoV particle contamination of common surfaces in airplane cabins, as well as in hospital air sources during outbreaks, via RNA quantification [7]. Importantly, Bonifait et al. reported that murine norovirus-1 (MNV-1) acts as a surrogate for Hu-NoVs, remaining infectious even after aerosolization, resulting in viable Hu-NoV particles attaching to environmental surfaces and that are capable of infecting individuals [6]. Indeed, the secondary contamination of surfaces by Hu-NoVs represents a significant route of infection, owing to their low infectious dose, long-term environmental persistence, and shedding by infected individuals.

The primary factors responsible for the bactericidal activity of slightly acidic electrolyzed water (SAEW) appear to be the presence of a high oxidation-reduction potential (ORP), the free available chlorine content (ACC), as well as the presence of hypochlorous acid (HOCl) and hypochlorite ions (OCl–) [8]. SAEW is generated by electrolyzing an aqueous solution of hydrochloric acid (HCl) or NaCl using a non-membrane electrolytic cell. The efficient form of free available chlorine compounds in SAEW is typically HOCl, with strong bactericidal efficacy in a pH range of 5.0–6.5 [8]. SAEW exhibits broad-spectrum antimicrobial effectiveness against a range of microorganisms, including non-enveloped viruses such as Hu-NoVs. Thus, the virucidal activities of SAEW against cultivable Hu-NoV surrogates (e.g., MNV) and Hu-NoV GII.4 Sydney in solution have been evaluated in several studies [9]. In spite of the widespread application and established effectiveness of SAEW against pathogenic microbials, there has not been a sufficient number of studies performed to assess its ability to decontaminate surfaces exposed to Hu-NoVs. This is largely owing to the fastidiousness of the reliable detection and quantitative assays for Hu-NoVs, based on RNA viability. To achieve a precise qualitative and quantitative analysis, Lee et al. demonstrated that the application of a magnetic bead separation (MBS) assessment combined with an intercalating dye, such as propidium monoazide (PMA), and reverse transcription-quantitative polymerase chain reactions (RT-qPCR) successfully and selectively detects viral particles, while distinguishing between intact and damaged particles [10]. Additionally, anionic surfactant treatments (e.g., sodium lauroyl sarcosinate (SLS)) [11] assist the penetration of inactivated viral capsids by PMA, thereby enhancing the distinction between noninfectious and infectious viruses. Hence, a combination of these techniques has the capacity to enhance the precise quantification of intact Hu-NoVs on environmental surfaces following exposure to chemical disinfectants.

Recently, among the various strategies for eliminating viral particles as human infection sources from environmental fomites, the spraying of chemical agents has proven successful for preventing the spread of infectious Hu-NoVs in healthcare and the public sector [12]. A significant advantage of spraying disinfectants in a mist from a machine is the dispersion effect achieved, facilitating the capture of airborne pathogens prior to rapidly settling on surfaces [13]. Hence, this strategy ensures that no chemical particles remain in the air after spraying the disinfectant, thus limiting the risk of inhalation by those administering the spray. Furthermore, this spraying technique has been applied in food industrial fields for the disinfection of fresh vegetables and fruits or the decontamination of surfaces in process lines and working areas [14]. According to the CDC [15], however, further research is required to verify the efficacy of chemical spraying for the elimination of Hu-NoV contamination. Specifically, more in-depth data are required to determine the optimal surface contact range for disinfectant sprays. Moreover, although many studies have been conducted with various chemical sprays, little is known regarding the optimum spraying treatments for the disinfection of Hu-NoVs using SAEW [16,17].

The specific objective of this study was to investigate the optimum virucidal activities with a SAEW spraying treatment on various inanimate surfaces. Moreover, a multivariate statistical assessment using a combination of response surface methodology (RSM) and a Box–Behnken design (BBD) was employed to define the optimal parameters and correlation between experimental data, including the process time, flow rate, and spray distance.

## 2. Materials and Methods

### 2.1. Viruses

Hu-NoV genogroup-I genotype-6 (Hu-NoV GI.6) and genogroup-II genotype-4 (Hu-NoV GII.4) were provided by the Waterborne Virus Bank (Seoul, Korea). In this study, the initial titer of Hu-NoV GI.6 and GII.4 suspension samples for virucidal effect test was approximately between 5.0 and 5.5 log10 genomic copies/500 µL. For the disinfection test, all Hu-NoV stock were prepared using 500 µL of the Hu-NoV sample. To prepare the virucidal test mixture, Hu-NoV (100 µL) was vortexed briefly with 400 µL of RNase-free water. Approximately 500-µL aliquots of the solution were stored at −80 °C.

### 2.2. Preparation of SAEW and SAEW Spraying Machine

SAEW was generated via electrolysis of 5.5% hydrochloric acid in a chamber without a membrane using electrolyzed water equipment (Purester m-Clean; Morinaga Engineering Co., Ltd., Tokyo, Japan) at a setting of 2.45 A and 21.5 V. The pH/ ORP values of the SAEW solution were measured using a dual-scale pH meter (Accumet model 25; Fisher Scientific Co., Fair Lawn, NJ, USA) equipped with ORP/pH electrodes. The production rate of SAEW solution was reached at a 9.8 L/min flow rate. The colorimetric method, using a digital chlorine test kit (RC-3F; Kasahara Chemical Instruments Corp., Saitama, Japan), was used to measure the ACC of SAEW. The initial SAEW had an ORP of 1123 mV, ACC of 33.22 ppm, and pH of 5.12. The prepared SAEWs were used immediately for analyses. The ORP, ACC, and pH of SAEW were measured in triplicate before the experiment. An electric ultra-low volume (ULV) sprayer (Atomer-2 RA04HS, JY Industry co, Seoul, Korea) was used to generate sprayed SAEW droplets (droplet size: 20–50 μm; maximum discharge capacity: 1.2 L/min).

### 2.3. Experimental Design of SAEW Spray Treatment Using an Electric ULV Sprayer

#### 2.3.1. Box–Behnken Design

RSM statistical analysis was conducted to determine correlations among experimental parameters and to determine the optimal parameters for spraying disinfectants to effectively decontaminate Hu-NoVs. The optimized experimental design was performed using BBD combined with RSM, as defined by Myers and Montgomery [18]. The study variables were the spraying rate, spraying time, and spraying distance for SAEW against Hu-NoVs. The experimental design using BBD, which is regarded as the most reliable design method, was conducted using the Minitab statistical software, version 20 (Systat Software Inc., San Jose, CA, USA). Herein, we selected three BBD levels. The three levels of BBD comprised a set of points located at the midpoint of each end and the replicated central point of the multidimensional cube to gain the second-order polynomial regression models. Individual variable experiments were designed to express the independent treatment conditions of spraying rate (100–300 mL/min), spraying time (2–10 s), and spraying distance (0.5–2.5 m). The three independent parameters were investigated at three levels: +1, high level; 0, midpoint to determine experimental error; −1, low level (Table 1).

To determine the efficacy of the three independent parameters on the SAEW spraying treatment for Hu-NoV decontamination, a quadratic regression model, as presented in Equation (1), was applied:

(1) $$Y=\beta_{0\;}+\sum_{i=1}^{k}\beta_{i}X_{i}+\sum_{i=1}^{k}\beta_{ii}X_{i}^{2}+\sum_{i_{i=1}}^{k}\sum_{j>1}^{k}\beta_{ij}X_{i}X_{j}+\varepsilon \;.$$

where *X_i_* and *X_j_* represent coded independent variables; *Y* is the response; *β*_0_, *β_i_*, *β_ii_*, and *β_ij_* are the constant coefficients of intercept, linear, interaction, and quadratic terms, respectively; and *ε* represents the error. The regression model significance was determined based on the adjusted coefficient of determination (R^2^ adj). Its statistical significance was verified with ANOVA, coefficient of multiple determination (R_2_), Fisher’s F test, and the lack of fit test in Minitab software. The 3D graphical plots of the Minitab software behavior, which was defined by the response surface, were used to describe the effective dispersion of SAEW spray droplets and the efficiency of the contact ratio to the target surface area.

#### 2.3.2. Analysis of the Dispersion Pattern of SAEW Sprayed Particles

**Figure 1:**
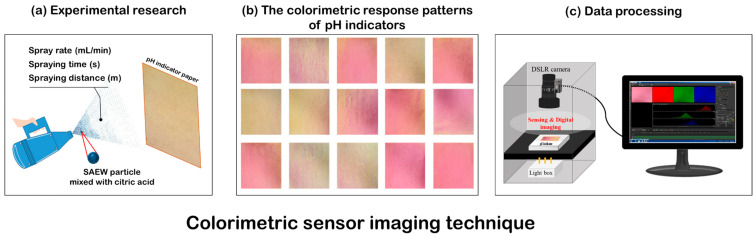
Schematic of the experimental design for optimized spraying conditions. Measurement of (**a**) dispersion and contact pattern of SAEW spray droplets, (**b**) colorimetric response patterns of pH indicators, and (**c**) SAEW spray droplets via a computational system.

To identify the dispersion and contact pattern of SAEW spray droplets via a computational system, an analytical technique based on a colorimetric sensing image, combined with pattern-recognition methods, was used (Figure 1).

Food-grade citric acid (Sigma-Aldrich, St. Louis, MO, USA) was added to lower the pH of the SAEW solution. The mixture increased the sensitivity of the pH-sensitive indicator (Universal pH Paper, Waterloo, ON, Canada) response. SAEW with citric acid and a pH-sensitive indicator was used to confirm the dispersion pattern of SAEW fogged droplets emitted from the electric ULV sprayer in the open atmosphere and to compare the relative quantitative values of SAEW in contact with the target surface, according to the spray conditions.

First, colorimetric sensor imaging was performed on SAEW spray-treated pH-sensitive indicator paper, which was placed in the open-frame stage of an online monitoring system coupled with NIS-Element software, which was developed for this work (Figure 1). One sheet of pH-sensitive indicator paper was positioned and fixed vertically on a laboratory workbench. The SAEW sprayer was activated according to the designated experimental conditions. The experimental treatment conditions, as designated by the BBD (Table 1), are shown in Table 2.

#### 2.3.3. Kinetic Parameters of the pH-Sensitive Indicator

L*, a*, and b* values for the color changes of the SAEW spray-treated pH-sensitive indicator paper were obtained using a CR-420 chromameter (Konica Minolta, Osaka, Japan). According to Giannakourou and Taoukis [19], based on the Hunter *L*, *a*, and *b* color, the chroma values were determined to efficiently quantify the total color difference of the pH-sensitive indicator, using the chroma value equation (Equation (2)):

(2) $$C=\sqrt{{\left(a^{*}\right)}^{2}+{\left(b^{*}\right)}^{2}}$$

Giannakourou and Taoukis [19] demonstrated that the normalized chroma value (*X_C_*) could be used as the response *X* of the colorimetric indicator, which, when plotted as a function of time, had a sigmoidal shape, similar to a Gaussian function (determined as *X* = 1 − exp[–(kt)^2^]). The normalization of the chroma value equation (Equation (3)) was expressed as follows:

(3) $$X_{C}=\frac{\left(C-C_{min}\right)}{\left(C_{max}-C_{min}\right)}$$

Finally, the color response values of the pH-sensitive indicators were expressed using the following linearized response equation (Equation (4)):

(4) $$F\left(X_{C}\right)=\sqrt{\ln\;\left[\frac{1}{\left(1-X_{C}\right)}\right]}$$

where F(*X_C_*) indicates the color response value.

#### 2.3.4. Pressure Distribution Measurement System

The pressure distribution, which corresponded to the spraying rate of the ULV sprayer set in this study, was measured using a large area flexible pressure-sensing integrated circuit (IC) board (Snowboard, 1.7 mm × 1.7 mm, Kitronyx Corp, Seoul, Korea). The Snowboard, a tactile sensor array, is an Arduino Leonardo-compatible board with integrated spray pressure and contact-sensor controllers. The spray pressure-sensing IC and Snowboard software (ForceLAB software, Kitronyx Corp) enabled visualization of the pressure load in any resistive matrix sensor. For the air pressure distribution measurement, the distance between the sensor and the nozzle of the electric ULV sprayer was 30 cm, while the average observed value of the experimental data acquired in real time was derived from pressure distribution, measuring for 1 min.

### 2.4. Surface Materials

In this study, various environmental surfaces were examined to determine the optimal spraying rate, spraying time, and spraying distance for Hu-NoV decontamination using the SAEW sprayer The four material types were polyvinyl chloride (PVC) (HDPE; Hangiltech Co., Seoul, Korea), stainless steel (Hyundai BNG steel, SUS-ANSI 306, Seoul, Korea), ceramic tile (Hankook Chinaware Co., Ltd., Seoul, Korea), and glass (SeoulYuri, Seoul, Korea), which are representative materials of most environmental fomites. Sheets of the four types of material were cut into 10 cm × 10 cm pieces. Prepared surface materials were disinfected by immersing them thoroughly in 50,000 ppm sodium hypochlorite, washing with sterilized water in an Ultrasonic Cleaner (Sigma-Aldrich) for 5 min, and rinsing with deionized water. Each dried plate sheet was then wrapped with UV radiation-treated aluminum foil, placed in a glass beaker, and autoclaved at 121 °C prior to the experiment.

### 2.5. Evaluation of the Virucidal Activity of SAEW Sprayed Droplets

Approximately 200 µL each of the two Hu-NoV suspensions was mixed with 190 µL of phosphate-buffered saline (PBS, pH 7.5, Sigma-Aldrich) and 10 µL of the Hu-NoV sample. The four types of prepared material surface were then inoculated with the viral suspension (approximately 6.60 log_10_ genomic copies/µL). To enable absorption of the inoculated Hu-NoV suspension on the surface, all agitated suspensions were incubated for 60 min at 18 ± 3 °C in a laminar flow hood. The virucidal activity of the SAEW spraying treatment was checked using a modified Quantitative Disk Carrier Test (ASTM E2197), a disinfectant testing protocol recognized worldwide. First, virucidal activity experiments examining spraying systems were performed in a 3.4 m^3^ chamber, in which air ventilation was blocked to facilitate full control of the airflow. The electric ULV sprayer was loaded with SAEW solution at 33.22 ppm ACC, pH 5.12, and 1123 mV of ORP and used to generate sprayed droplets with a size of approximately 30 ± 10 μm at a 100–300 mL/min spraying rate. The individual contaminated surfaces were sprayed with SAEW under the following conditions: spraying rate (100–300 mL/min), spraying time (2–10 s), and spraying distance (0.5–2.5 m). Thereafter, the disinfected surface was left for 5, 10, 20, and 30 min to allow time for the SAEW droplets to inactivate viruses on the exposed surfaces. Triplicate spraying experiments were conducted for the evaluation of virucidal activity.

### 2.6. Microbiological Analyses

#### 2.6.1. Recovery of Hu-NoVs

Elution, concentration, and optimal quantification of Hu-NoVs from individual SAEW spray-disinfected surface samples were determined following the methodology of previous studies [20]. The optimized quantitative assay for elution and concentration of Hu-NoV GI.6 and GII.4 is summarized in the flow diagram (Figure S1). Immediately following SAEW spray disinfection, an Enviro-Max Environmental sampling cotton swab kit (Puritan Medical Products Company LLC, Guilford, ME, USA) was used to recover Hu-NoV particles from each surface sample. Each surface was swabbed diagonally, vertically, and horizontally on both sides of the cotton swab, 15 times in each direction. Subsequently, Hu-NoV particles were eluted using cotton swabs by repeatedly immersing in 20 mL of 0.14 M NaCl-0.05 M glycine (pH 7.0) for 5 min in the transport tube at 18 ± 3 °C with constant shaking (approximately 50 rpm). Each was then thoroughly mixed with a vortex machine for 60 s. Subsequently, cotton swabs were squeezed against the inside wall of the tube, to release all liquid. Each eluate was placed into a 50 mL conical tube. Approximately 20 mL of the secondary suspension was added to the 20 mL of primary elution suspension.

#### 2.6.2. Optimized Quantification of Hu-NoVs

Hu-NoV particles were concentrated using the MBS technique. For the MBS assay, 100 μL of magnetic bead suspension (final concentration: 10 mg/mL) was added to 40 mL of the viral mixture suspension and agitated for 1 h at 18 ± 3 °C. Magnetic beads with captured Hu-NoV particles were isolated using a LifeSep magnetic separation stand (Sigma-Aldrich), and resuspended with 140 µL of PBS. Following virucidal inactivation, RNA isolation and analysis were immediately conducted to avoid the effects induced by sample freezing.

Optimized quantification assays of viral particles from the SAEW spray-disinfected Hu-NoVs were performed as previously reported. Specifically, an MBS/RT-qPCR assay was conducted following pretreatment with combined SLS (Sigma-Aldrich) and PMA (MBS/PMA/SLS/RT-qPCR) [11]. This method provides optimal conditions for quantification, while conferring minimal damage to intact Hu-NoV particles. Each viral suspension treated with PMA dye was concentrated using MBS, immediately mixed further with 0.2 mM PMA, and incubated in the dark at 4 °C for 5 min, to allow dye penetration. A high-power LED light (45-W lamp) in a photo-activation system (PhAST Blue; GenIUL, Spain) was then used for irradiation of samples at a wavelength of 460 nm at 4 °C for 15 min.

#### 2.6.3. Viral RNA Extraction and Quantitative RT-qPCR

The Hu-NoV GI.6 and GII.4 RNA were purified using a QIAamp Min-Elute virus spin kit (Qiagen, Hilden, Germany), following the manufacturer’s instructions. Approximately 60 mL of AVE buffer was used to elute Hu-NoV viral RNA, which was then used immediately to avoid RNA degradation associated with viral RNA freezing. Approximately 5 mL aliquots of each RNA suspension were subjected to one-step RT-qPCR using a QuantiTect Probe RT-PCR kit (Qiagen) and Real-Time PCR System (Applied Biosystems 7500 Fast system, Foster City, CA, USA).

For Hu-NoV GI.6 and GII4, one-step RT-qPCR was conducted using 5 μL of viral RNA extracted from a total volume of 20 μL. The following cycling parameters were used: 50 °C for 600 s, denaturation at 95 °C for 300 s, 45 cycles of amplification with denaturation at 95 °C for 10 s, and combined annealing and extension at 60 °C for 30 s. For Hu-NoV GI-6, the TaqMan probe (JJV1P) sequence was FAM 5′-TGT∙GGA∙CAG∙GAG∙ATC∙GCA∙ATC∙TC-3′ BHQ [10]. Hu-NoV GI primer (10 pmol each) sequences were 5′-JJV1^R^ TCC∙TTA∙GAC∙GCC∙ATC∙ATC∙AT-3′ and JJV1^F^ 5′-GCC∙ATG∙TTC∙CGI∙TGG∙ATG-3′. These primers were used to amplify a 96-base pair (bp) fragment of the Hu-NoV GI polymerase gene. For Hu-NoV GII.4, the TaqMan probe (Ring^2^, 10 mM) was FAM: 50-TGG∙GAG∙GGC∙GAT∙CGC∙AAT∙CT-30 BHQ. The Hu-NoV GII.4 primer sequences (10 mM each) were COG2^R^: 50-TCG∙ACG∙CCA∙TCT∙TCA∙TTC∙ACA-30 and COG2^F^: 50-CAR∙GAR∙BCN∙ATG∙TTY∙AGR∙ATG∙AG-30, which were used to amplify a 122-bp fragment of the Hu-NoV GII.4 [10].

### 2.7. Statistical Analyses

Trials of all experiments were carried out in triplicate. For the RT-qPCR assay, the experimental data were plotted using Minitab^®^ statistical software and were expressed as log_10_ genomic copies/μL. For statistical analysis, the one-way ANOVA test in Minitab^®^ statistical software and Duncan’s multiple range test were used to compare differences between mean values. Moreover, ANOVA, in the Minitab^®^ statistical software, was used to compare differences between mean values. A *p*-value < 0.05 was defined as statistically significant. The experimental results were denoted as log_10_ genomic copies/μL. Regression analysis was conducted using the Sigma Plot software system, version 14.0 (San Jose, CA, USA).

## 3. Results and Discussion

### 3.1. Pressure Distribution Measurement System

The higher the pressure at the electric ULV sprayer inlet, the smaller the size of the spray particles, which has a significant effect on spray dispersion [21]. Moreover, a typical ULV sprayer produces thousands of droplets each second. Therefore, it is impractical to select the number of particles based on arithmetic calculations as a parameter for the optimum condition of the sprayer. Figure S2 presents a visualization of the pressure of the sprayer nozzle based on the treatment condition of the spraying rate (100, 200, and 300 mL/min) derived from BBD.

### 3.2. Changes in Physicochemical of SAEW after Spraying Treatment

The physicochemical properties of the disinfectant can be altered owing to a rapid decrease in the cross-sectional area of SAEW droplets (average particle size 20–50 μm aerosol mist) during the spraying process [22]. Table 3 shows the changes in physicochemical pH, ORP, and ACC of the original SAEW and sprayed SAEW. Overall, the pH and ACC of SAEW changed significantly with increasing distance between the spray nozzle and contact surface, whereas the ORP values remained stable at 1.5 m. According to Zhao et al., spraying treatment increases the pH values by approximately 1.0 when the ACC is decreased by approximately 70%, making the SAEW slightly more basic [23]; it is speculated that the physiochemical properties of SAEW are altered owing to the evaporation of chlorine gas. However, despite the observed changes in the pH and ACC values of the sprayed SAEW, all tested SAEW samples fell within the range of typical SAEW properties (20 to 80 mg/L ACC, approximately 1000 mV ORP, and 5.0 to 6.0 near-neutral pH). Hence, sprayed SAEW, with altered shape and smaller droplets, maintains its virucidal effect.

### 3.3. Model Development and Statistical Analysis

The BBD-based experimental results are presented in Table 4 along with a comparison of the predicted and observed color response values (F(*X_C_*)) after spraying the SAEW-citric acid mixture onto pH-sensitive indicator paper. Figure S3 shows a visualization of pH-sensitive indicator papers, one of which was randomly selected from each spray treatment trial. Additionally, it is possible to observe changes in the R (red), G (green), and B (blue) values of the pH-sensitive indicator papers, according to the dispersion pattern. The regression equation (Equation (5)), used to compare changes in the F(*X_C_*) of the pH-sensitive indicator paper in terms of the coded values of variables is as follows:

F(*X_C_*) = −0.373 + 0.00724 *X*_1_ + 0.0337 *X*_2_ + 0.657 *X*_3_ + 0.000007 *X*_1_*X*_1_ + 0.00223 *X*_2_*X*_2_ − 0.1614 *X*_3_*X*_3_ + 0.000714 *X*_1_*X*_2_ − 0.003173 *X*_1_*X*_3_ − 0.0272 *X*_2_*X*_3_ (5)

where *X*_1_, *X*_2_, and *X*_3_ are the uncoded values of the spraying rate (mL/min), spraying time (s), and spraying distance (m), respectively.

The color response value parameters for the dispersion pattern of SAEW sprayed particles were estimated. According to the ANOVA results (Table 4), the F-value < Prob was less than 0.05 with an F-value of 39.11, indicating an acceptable model fit and that the three variables had a prominent effect on the dispersion pattern of SAEW sprayed particles. Both the F- and *p*-values demonstrate the significance of variable coefficients. That is, lower *p*-values and higher F-values indicate that a more important contribution was made by the corresponding model term toward the response variable [24]. As a result, the order in which the test variables affected the response was as follows: spraying distance (m) > spraying time (s) > rate (mL/min). With respect to the dispersion pattern of SAEW sprayed particles, the spraying distance (m) had the most significant influence on the color response parameters (F-value: 161.47), whereas both the spraying rate (mL/min) and spraying time (s) had a less significant effect (F-value: 66.64 and 78.17, respectively). The parameters that impacted the interaction strength were the spraying rate and spraying distance (*p*-value: 0.006). However, the relationship between the spraying distance and time was not significant (*p*-value: 0.178), suggesting that the uniformity of sprayed SAEW particle dispersion does not impact the response, even if the spray time increases with increased spray distance. Hence, the spraying distance and rate have a greater impact than spraying time on ensuring an even diffusion of SAEW spray particles on the target surface. That is, the efficiency of SAEW spray particle dispersion can be increased by regulating spraying distance and rate.

**Figure 2:**
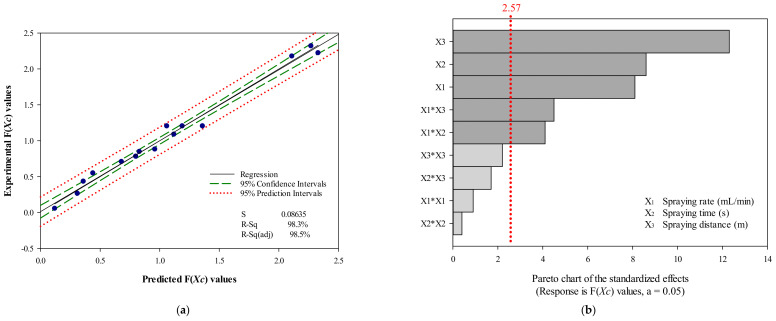
(**a**) Fitted line plot for the predicted and experimental values of the color response value (F(*X_C_*)), (**b**) standardized Pareto chart for the color response value (F(*X_C_*)).

If the *p*-value for the ‘lack of fit’ is <0.05, the predicted model is inadequate, whereas if it is greater than 0.05, the obtained model is appropriate [25]. In our study, the *p*-value related to lack of fit in the obtained model was 0.637, and the model obtained from the ANOVA procedure was found to be acceptable [25]. The significance of the selected quadratic model was calculated using regression coefficient (R^2^) values, which revealed high coefficients (0.986) for the uniformity of SAEW spray particle dispersion obtained from F(*X_C_*) values. Furthermore, the high R^2^ obtained for the comparison of the predicted F(*X_C_*) and experimental F(*X_C_*) values (R^2^ = 0.983; Figure 2a) demonstrated that this quadratic model could be applied to predict optimized experimental conditions for a SAEW spray disinfection treatment. The R^2^ (0.983) for the SAEW spray particle dispersion implied that only 1.7% variation could not be explained by the obtained model.

In addition, the Pareto analysis (Figure 2b) identified the SAEW disinfection variables (spraying rate, time, and distance) that have the greatest impact on the efficiency and reliability of a SAEW spray disinfection treatment, which were illustrated and selected based on the data from the experimental results in Table 4. According to Secula et al., the Pareto chart, demonstrating the absolute value of the standardized influencing factors, is used to define the importance and magnitude of the effects among the independent parameter effect, second-order effect, and interaction effect [26]. Moreover, the Pareto chart indicates the significance of each variable investigated in the experimental data and illustrates the primary effects of the factors, to be ranked in order of their significance [25]. The vertical line in the Pareto chart shows a table value of 2.57 with a 95% confidence level, whereas the horizontal bar chart indicates the calculated t-values. According to this comprehensive statistical analysis, a quadratic model could be applied to predict the optimized experimental conditions for SAEW spray treatment.

### 3.4. Response Surface Plots for Parameter Optimization

**Figure 3:**
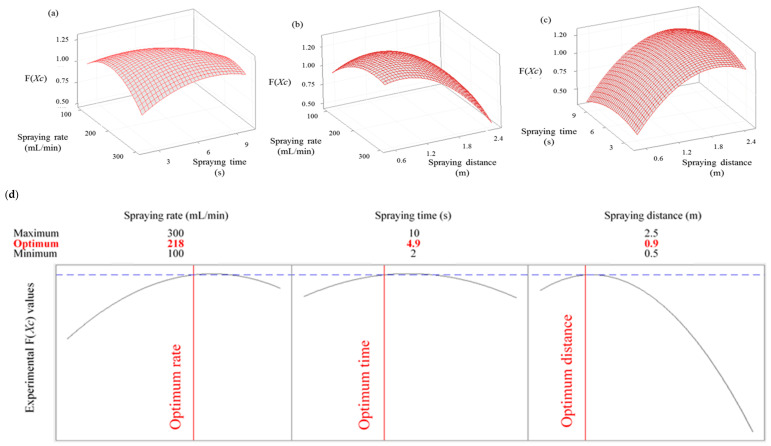
Three-dimensional response surface plots for the color response values (F(*X_C_*)) from the dispersion and contact pattern of SAEW spray droplets: (**a**) spraying rate and spraying time, (**b**) spraying rate and spraying distance, (**c**) spraying time and spraying time, (**d**) optimization results for slightly acidic electrolyzed water spray under different conditions, according to the response surface methodology. Multiple optimization graphs of experimental color response values (F(*X_C_*)).

To establish optimized parameters of SAEW spray disinfection treatment, a 3D response surface plot was used to predict the interaction between a pair of factors, while fixing all other parameters. The interactions between spraying rate and spraying time, spraying rate and spraying distance, and spraying time and spraying distance for the dispersion of SAEW sprayed particles using an electric ULV sprayer are illustrated in Figure 3a–c, whereas Figure 3d presents a graph of the optimal conditions for the three parameters with maximum color response values (F(*X_C_*)), indicating that the spray particles were effectively dispersed. The optimized F(*X_C_*) values for the three process parameters were acquired using the mathematical multiple optimization method; namely, a spraying rate of 218 mL/min, spraying time of 4.9 s, and spraying distance of 0.9 m. These optimum parameters provided a predicted F(*X_C_*) of 1.3079 and a specific F(*X_C_*) of 1.311 for the dispersion pattern of SAEW sprayed particles using an electric ULV sprayer. Both results are the same as the predicted values, within numerical error, confirming the suitability and validity of the predicted model. When considering the efficiency of spray disinfection treatment, it has been recognized that the amount of time that the sprayed disinfectant remains on the contaminant (i.e., contact time) is the most important factor [27]. Indeed, contact time is clearly indicated on the label of liquid disinfectants registered with the US Environmental Protection Agency (EPA) [28]. Thus, spray sterilization can be defined as effective only when direct contact between the disinfectant and microorganisms occurs for a sufficient length of time. The lack of scientific data to support the required contact time between disinfectants and microorganisms may account for the persistent issues regarding the reliability of spray sterilization techniques. As such, we believe that our findings advance the current understanding regarding the optimal conditions associated with the application of SAEW mist to contaminants on environmental surfaces. Moreover, we have demonstrated that a uniform and complete dispersion of the disinfectant particles sprayed from a nebulizer ensures sufficient contact time.

### 3.5. Validation of MBS/PMA/SLS Pretreatment Combined with RT-qPCR Assay

In the present study, MBS/PMA/SLS and RT-qPCR were the methods employed to quantitatively and qualitatively investigate the viability of Hu-NoVs following disinfection with SAEW. From the point of view of false-positive outcomes caused by the nonspecific amplification of inactivated and infectious viruses, the RT-qPCR assay produced cycle threshold (Ct) values for both Hu-NoV GI.6 and GII.4 after treatment with SAEW (ORP of 1123 mV, pH of 5.12, and ACC of 33.22 ppm), and both Hu-NoVs were amplified at a value of approximately 40 Ct by MBS/PMA/SLS/RT-qPCR (Figure S4). Our findings demonstrated that RT-qPCR, without PMA-SLS, amplified target viruses after SAEW treatment, resulting in false-positive outcomes, despite an adequate inactivation treatment.

Although RT-qPCR assays are widely used to quantify targeted viral particles, owing to their short assay time, sensitivity, and specificity, certain technical obstacles exist when applying nucleic acid-based detection assays to the assessment of viral particles after chemical inactivation. One such obstacle is their inability to distinguish noninfectious from infectious viruses in disinfected viral samples. Conversely, specific MBS has been shown to effectively help avoid these pitfalls [29]. Indeed, MBS technology has been reported to increase virus concentration and detection sensitivity [30,31]. In addition, numerous studies have reported that pretreatment of samples with intercalating dyes (PMA or ethidium monoazide) can improve the discrimination of intact viral particles. As such, these more advanced analysis techniques are regularly adopted to eliminate the false-positive results caused by nonspecific binding to magnetic beads [32,33,34]. Furthermore, SLS, an anionic surfactant, has been shown to facilitate the entry of intercalating dyes into injured viral capsid proteins, thereby enhancing the differentiation of noninfectious and infectious viral particles [35,36]. Hence, MBS/PMA/SLS treatment combined with RT-qPCR, as performed in this study, is believed to be a suitable and reliable strategy for distinguishing among dead, damaged, and intact viral particles following SAEW spray treatment.

### 3.6. Virucidal Effect of the Optimal Spraying Treatment

**Figure 4:**
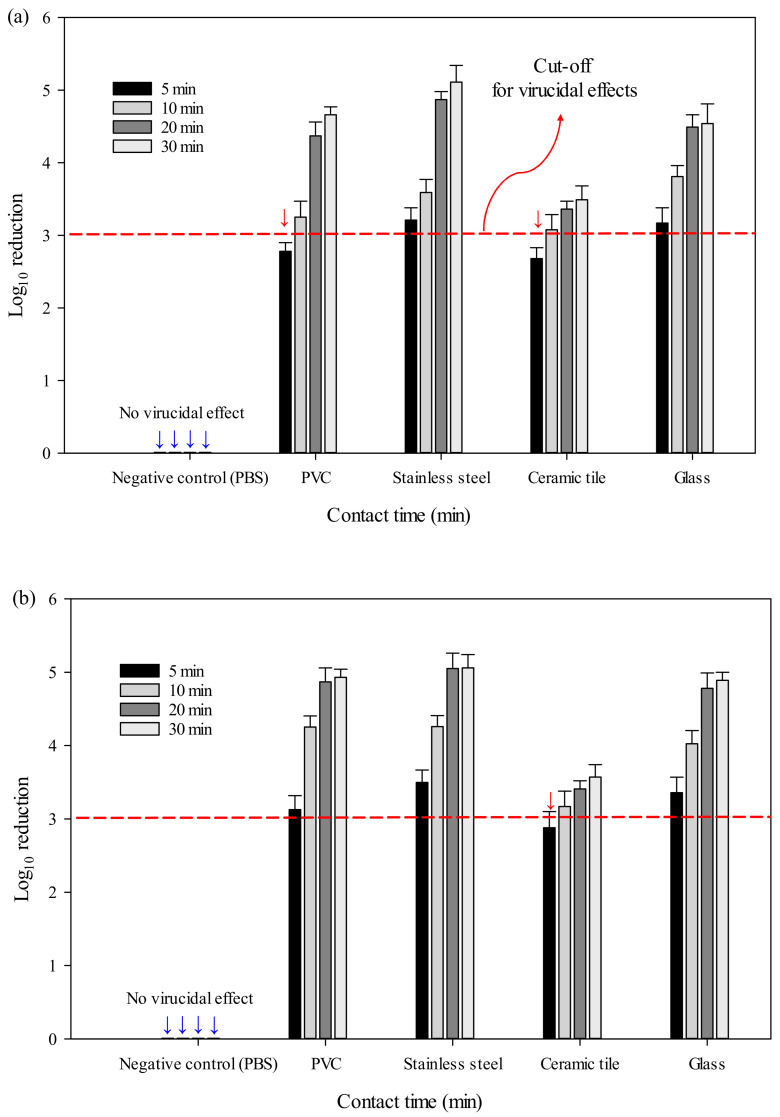
Reduction values of (**a**) Hu-NoV GI.6 and (**b**) GII.4 titers after disinfection with slightly acidic electrolyzed water spray under optimal treatment conditions on PVC, stainless steel, ceramic tile, and glass surfaces.

To evaluate the validity of the given quadratic model based on the BBD, virucidal tests were performed under optimized conditions (Figure 4). For Hu-NoV GI.6, the virucidal efficacies on PVC, stainless steel, ceramic tile, and glass surfaces at optimum conditions for a 30-min treatment were 4.66 ± 0.11, 5.11 ± 0.23, 3.57 ± 0.19, and 4.54 ± 0.27 log reduction, respectively. Similarly, Hu-NoV GII.4, SAEW disinfection under optimal conditions with a 30-min treatment had log reduction values of 4.89 ± 0.31, 5.06 ± 0.18, 3.49 ± 0.22, and 4.89 ± 0.27 on the four surfaces, respectively. More specifically, following exposure to sprayed SAEW virucidal treatment for more than 5 min, a greater than 3 log viral reduction was achieved in most treatment groups, apart for Hu-NoV GI.6 inoculated on PVC and ceramic tile surfaces and Hu-NoV GII.4 inoculated on ceramic tile surfaces. Furthermore, sprayed PBS droplets, which served as the negative control, elicited no inactivation effect against either Hu-NoV on the four surfaces.

For NoVs on various material-specific surfaces, the magnitude of virus reduction was dependent on the exposure time after spray treatment. These results indicate that they were derived by considering the norovirus control effect and the economic effect of spray sterilization. Interestingly, the virucidal effect of optimal SAEW disinfection on both Hu-NoVs was lower on ceramic tiles than on other surfaces, likely owing to the high water absorption of ceramic tiles, which is believed to obstruct the virucidal effect by not allowing sufficient time for the spray particles of SAEW to react with the viral particles. Marks et al. suggested that a chemical disinfectant could effectively disinfect surfaces carrying viral particles if it causes a 3 log10 value reduction in virus population, taking into consideration the amount of virus shed into the environment [37]. Hence, our findings demonstrated that SAEW disinfection treatment under optimal conditions for 10 min or more achieves acceptable levels of Hu-NoV decontamination on four common surfaces.

As the COVID-19 pandemic continues, there is a growing consensus that sufficient disinfection and the prevention of surfaces from being contaminated with pathogenic viral particles is essential, to slow the spread of this virus in complex multi-facilities, schools, hotels, nursing homes, and hospitals. ULV sprayer disinfection represents an effective approach for preventing surface-based viral particle transmission. However, although this method offers certain benefits, the efficiency of other techniques, particularly for the inactivation of Hu-NoVs on various surfaces, has not been adequately investigated. In fact, to date, a comprehensive assessment of the optimal models for ULV sprayer disinfectants is lacking, particularly regarding the optimal physics of these sprayers and surface characteristics to ensure inactivation of Hu-NoVs. Nasr et al. suggested that disinfection efficiency is affected by several parameters, including spray droplet size, spray pressure, disinfectant contact time, disinfectant droplet distribution, and distance from surfaces; all of which must be considered when seeking to verify the effectiveness of spray disinfection [38].

## 4. Conclusions

The fomite transmission of Hu-NoVs, a common human pathogen, requires decontamination techniques for prevention and control. Optimized experimental conditions for SAEW spraying (spraying rate: 218 mL/min, spraying time: 4.9 s, and spraying distance: 0.9 m) show that SAEW spray disinfection (ORP of 1123 mV, pH range of 5.12, and ACC of 33.22 ppm) is efficient for the inactivation of Hu-NoVs on fomite surfaces. We believe that the utilization of a ULV sprayer loaded with SAEW can provide a successful decontamination of surfaces. Furthermore, this technique enables the control of Hu-NoV and prevents it from being transmitted via environmental surface exposure. However, our experimental results are not representative of all commercially available electric ULV sprayers, and the optimal disinfection conditions for each spray sterilizer product may vary. Thus, virucidal evaluation of various electric ULV sprayer products must be performed in follow-up studies. Collectively, the findings of this study may have a significant impact on the strategic control practices of infectious disease transmission and in the prevention of Hu-NoV outbreaks.

## Figures and Tables

**Table 1:** Box–Behnken design matrix parameters and levels used to assess slightly acidic electrolyzed water spraying disinfection conditions.

Parameter	Symbol	Levels		
		Low (−1)	Intermediate (0)	High (+1)
Spraying rate (mL/min)	X_1_	100	200	300
Spraying time (s)	X_2_	2	6	10
Spraying distance (m)	X_3_	0.5	1.5	2.5

**Table 2:** Three-parameter BBD matrix with predicted and observed color response values (F(*X_C_*)).

Run	Coded Value			Actual Value			F(*X_C_*)	
	*X*_1_	*X*_2_	*X*_3_	*X*_1_	*X*_2_	*X*_3_	Observed	Predicted
1	0	0	0	200	6	1.5	1.36	1. 38
2	0	−1	1	200	2	2.5	0.68	0.49
3	−1	0	−1	100	6	0.5	0.96	0.81
4	0	1	1	200	10	2.5	0.31	0.33
5	1	0	1	300	6	2.5	0.12	0.25
6	1	−1	0	300	2	1.5	0.83	0.87
7	−1	−1	0	100	2	1.5	0.80	0.96
8	−1	1	0	100	10	1.5	0.73	0.68
9	0	−1	−1	200	2	0.5	1.12	1.09
10	0	1	−1	200	10	0.5	0.99	1.17
11	0	0	0	200	6	1.5	1.06	1.17
12	−1	0	1	100	6	2.5	0.36	0.37
13	1	0	−1	300	6	0.5	1.27	1.25
14	1	1	0	300	10	1.5	1.25	1.08
15	0	0	0	200	6	1.5	1.19	1.21

**Table 3:** Physicochemical properties of slightly acidic electrolyzed water samples collected after spraying.

Property	Original SAEW	SAEW Captured from a Distance (m)				
		0.5	1	1.5	2	2.5
pH	5.12 ± 0.01 ^a^	5.31 ± 0.02 ^b^	5.38 ± 0.02 ^bc^	5.45 ± 0.03 ^c^	5.59 ± 0.01 ^d^	5.61 ± 0.01 ^d^
ORP (mV)	1123 ± 7 ^a^	1026 ± 5 ^a^	1007 ± 13 ^a^	1001 ± 9 ^a^	983 ± 11 ^b^	981 ± 7 ^b^
ACC (ppm)	34.22 ± 0.31 ^a^	33.58 ± 0.37 ^a^	32.93 ± 0.34 ^ab^	30.91 ± 0.22 ^b^	29.18 ± 0.29 ^c^	29.17 ± 0.22 ^d^

^a–d^ Different lower-case letters indicate statistically significant differences between SAEW samples for the same physicochemical properties, *p* < 0.05.

**Table 4:** Matrix design results for the experiments performed according to the Box–Behnken experimental design for the color response values (F(*Xc*)) of the sprayed SAEW droplet dispersion and contact pattern.

Source	DF ^(1)^	F(*X_C_*)			
		Adj SS ^(2)^	Adj MS ^(3)^	F-Value	*p*-Value
Regression	9	6.82322	0.75814	39.11	0.000
e	3	5.93777	1.97926	102.09	0.000
*X*_1_	1	1.29191	1.29191	66.64	0.000
*X*_2_	1	1.51548	1.51548	78.17	0.000
*X*_3_	1	3.13038	3.13038	161.47	0.000
Square	3	0.10930	0.03643	1.88	0.251
*X*_1_ *X*_1_	1	0.01733	0.01733	0.89	0.388
*X*_2_ *X*_2_	1	0.00472	0.00472	0.24	0.643
*X*_3_ *X*_3_	1	0.09621	0.09621	4.96	0.076
Interaction	3	0.77615	0.25872	13.34	0.008
*X*_1_ *X*_2_	1	0.32596	0.32596	16.81	0.009
*X*_1_ *X*_3_	1	0.40271	0.40271	20.77	0.006
*X*_2_ *X*_3_	1	0.04749	0.04749	2.45	0.178
Residual Error	5	0.09693	0.01939		
Lack of Fit	3	0.04933	0.01644	0.69	0.637
Pure Error	2	0.04761	0.02380		
*R* ^2^		98.60			

^(1)^ DF, degrees of freedom; ^(2)^ Adj SS, adjusted sum of square; ^(3)^ Adj MS, adjusted mean square.

## Data Availability

The datasets used and/or analyzed during the current study are available from the corresponding author on reasonable request.

## Supplementary Materials

The following are available online at https://www.mdpi.com/article/10.3390/ijerph181910183/s1, Figure S1: Flow diagram of the analytical methods used for the quantitative evaluation of the virucidal efficacy for Hu-NoVs.; Figure S2: Visualization of the sprayer nozzle pressure based on the treatment condition of the spraying rate (a) 100, (b) 200, and (c) 300 mL/min.; Figure S3: Visualization of the dispersion and contact pattern of SAEW spray droplets based on BBD treatment conditions (15 run set).; Figure S4: Comparison of RT-qPCR assay with MBS/PMA/SLS/RT-qPCR for the evaluation of inactivation of disinfected NoV GI.6 and GII.4.
