# Supplementary material for: Optimizing Operating Parameters of Electric Ultra-Low Volume Sprayer with Slightly Acidic Electrolyzed Solution for Efficient Virucidal Activity on Environmental Surfaces
Source: Int J Environ Res Public Health. 2021 Sep 28;18(19):10183. doi: 10.3390/ijerph181910183 (PMC8508509; doi:10.3390/ijerph181910183)
Supplement: Supplementary file 1 [file ijerph-18-10183-s001.zip › ijerph-1365389-supplementary.pdf]

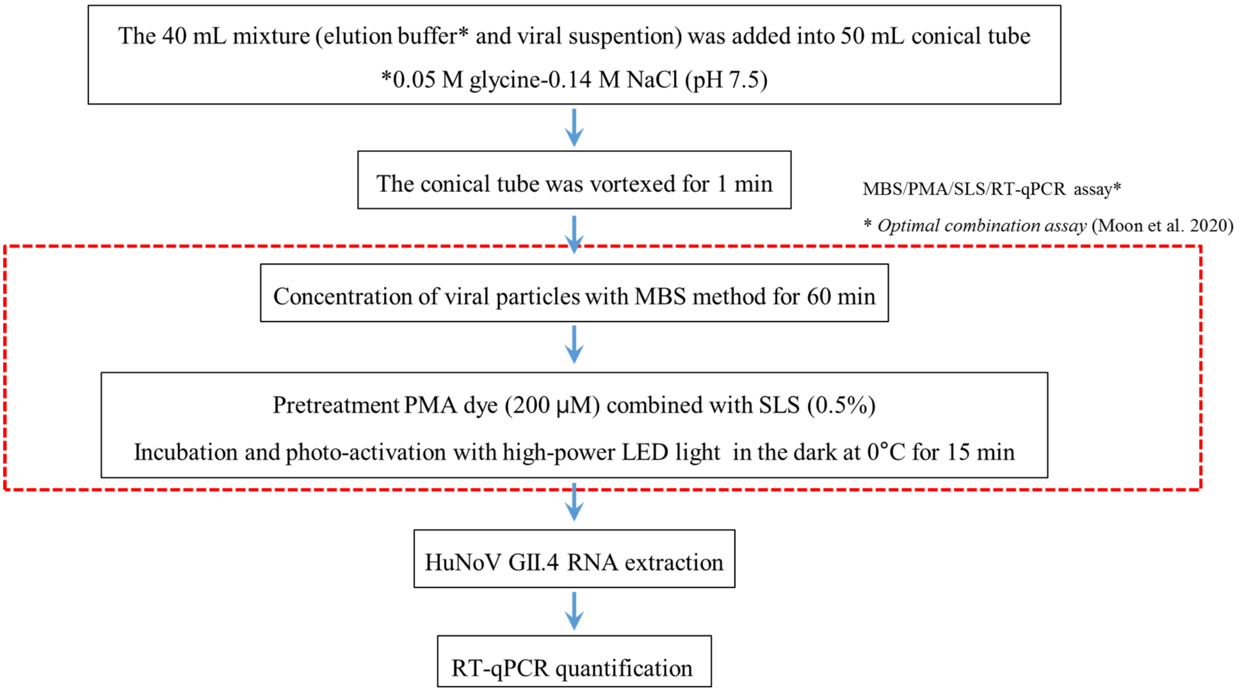

Figure S1. Flow diagram of the analytical methods used for the quantitative evaluation of the virucidal efficacy for HuNoVs.

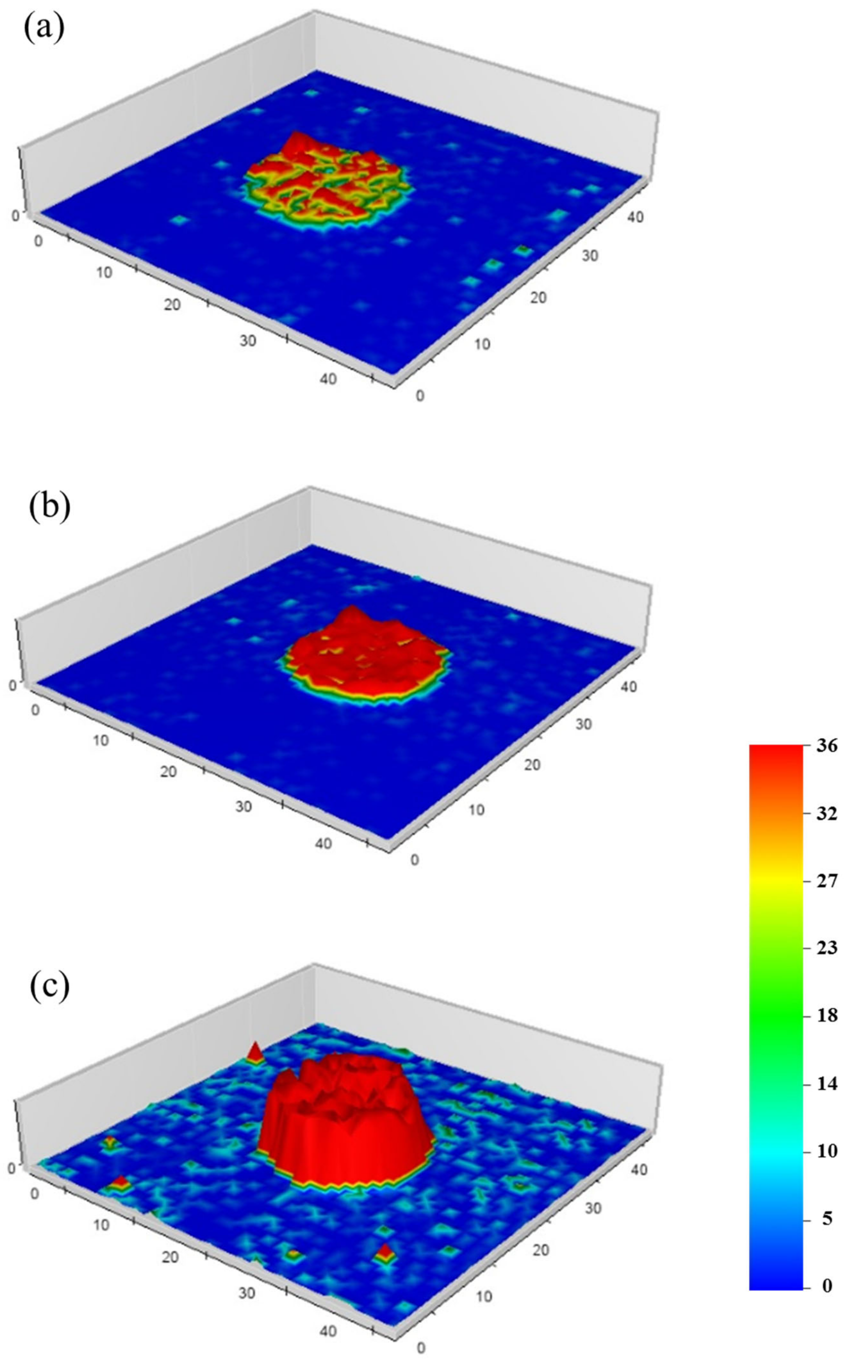

Figure S2. Visualization of the sprayer nozzle pressure based on the treatment condition of the spraying rate (**a**) 100, (**b**) 200, and (**c**) 300 mL/min.

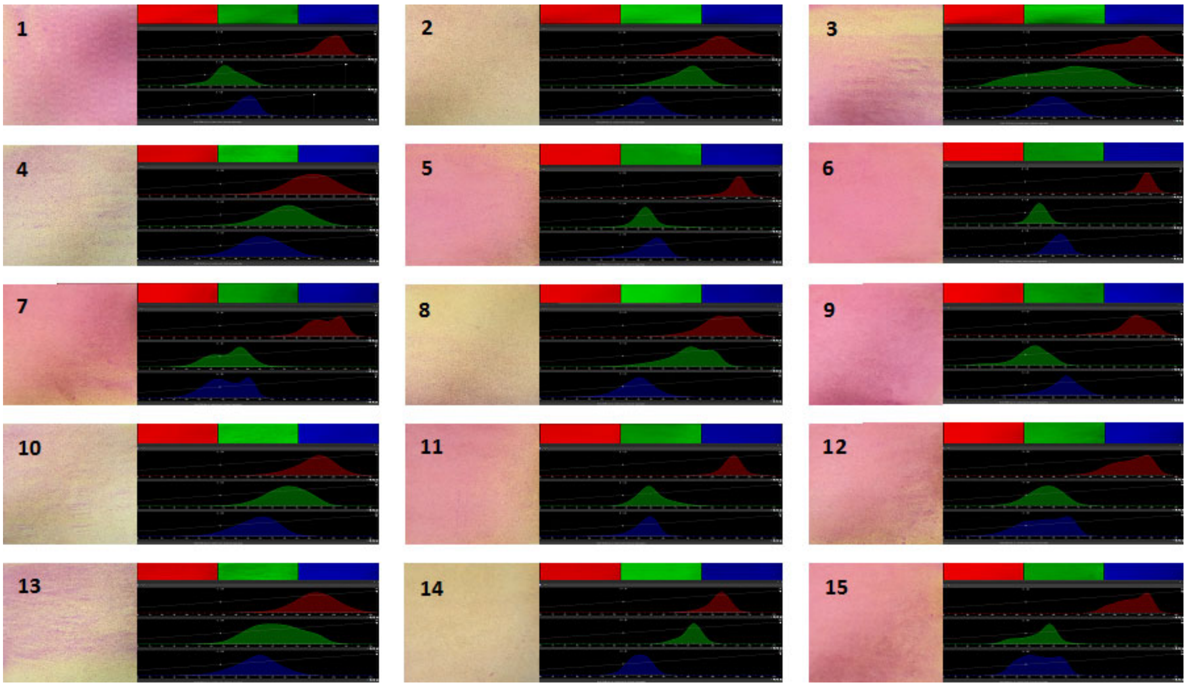

Figure S3. Visualization of the dispersion and contact pattern of SAEW spray droplets based on BBD treatment conditions (15 run set).

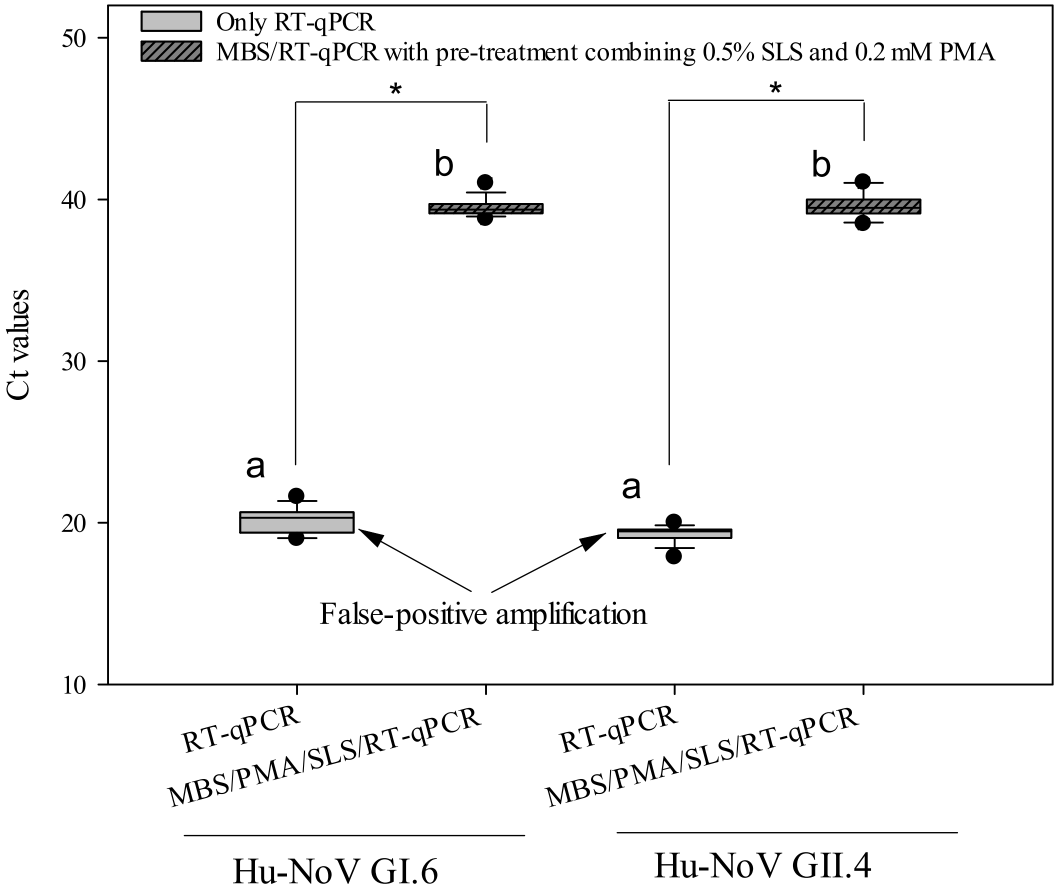

Figure S4. Comparison of RT-qPCR assay with MBS/PMA/SLS/RT-qPCR for the evaluation of inactivation of disinfected NoV GI.6 and GII.4.
